# Estimation of the stage-wise costs of breast cancer in Germany using a modeling approach

**DOI:** 10.3389/fpubh.2022.946544

**Published:** 2023-01-06

**Authors:** Shah Alam Khan, Karla Hernandez-Villafuerte, Diego Hernandez, Michael Schlander

**Affiliations:** ^1^Division of Health Economics, German Cancer Research Center (DKFZ), Heidelberg, Germany; ^2^Medical Faculty Mannheim, University of Heidelberg, Mannheim, Germany; ^3^Faculty of Economics and Social Sciences, Alfred Weber Institute (AWI), University of Heidelberg, Heidelberg, Germany

**Keywords:** breast cancer, stage-wise costs, direct medical costs, modeling, review, stage-specific costs

## Abstract

Breast cancer (BC) is a heterogeneous disease representing a substantial economic burden. In order to develop policies that successfully decrease this burden, the factors affecting costs need to be fully understood. Evidence suggests that early-stage BC has a lower cost than a late stage BC. We aim to provide conservative estimates of BC's stage-wise medical costs from German healthcare and the payer's perspective. To this end, we conducted a literature review of articles evaluating stage-wise costs of BC in Germany through PubMed, Web of Science, and Econ Lit databases supplemented by Google Scholar. We developed a decision tree model to estimate BC-related medical costs in Germany using available treatment and cost information. The review generated seven studies; none estimated the stage-wise costs of BC. The studies were classified into two groups: case scenarios (five studies) and two studies based on administrative data. The first sickness funds data study (Gruber et al., 2012) used information from the year 1999 to approach BC attributable cost; their results suggest a range between €3,929 and €11,787 depending on age. The second study (Kreis, Plöthner et al., 2020) used 2011–2014 data and suggested an initial phase incremental cost of €21,499, an intermediate phase cost of €2,620, and a terminal phase cost of €34,513 per incident case. Our decision tree model-based BC stage-wise cost estimates were €21,523 for stage I, €25,679 for stage II, €30,156 for stage III, and €42,086 for stage IV. Alternatively, the modeled cost estimates are €20,284 for the initial phase of care, €851 for the intermediate phase of care, and €34,963 for the terminal phase of care. Our estimates for phases of care are consistent with recent German estimates provided by Kreis et al. Furthermore, the data collected by sickness funds are collected primarily for reimbursement purposes, where the German ICD-10 classification system defines a cancer diagnosis. As a result, claims data lack the clinical information necessary to understand stage-wise BC costs. Our model-based estimates fill the gap and inform future economic evaluations of BC interventions.

## 1. Introduction

Breast cancer (BC) has a tremendous cost to healthcare systems, payers, patients, and society (1). According to the German Bureau of Federal Statistics, direct medical costs related to BC were around €2.17 billion in 2015, surpassing that of lung cancer (2), with around 67,000 new cases diagnosed and more than 18,000 deaths reported every year (3). Nearly one in eight women in Germany develop BC during their lives, and three in every ten women with BC are younger than 55 years of age (3). Mortality is considerably lower in women diagnosed in earlier stages than in advanced stages (4). Around 90% of the women diagnosed at an earlier stage survive for at least 5 years compa**r**ed to 15% at a late stage (5). Therefore, early detection through BC screening has the potential to save lives. Prominently, BC screening in Germany has significantly reduced late-stage incidence (6). Nevertheless, based on our analysis of the German cancer registry data, there is a lot more room for improvement since a considerable proportion (18%) of new BC patients are still diagnosed in the advanced stages (III and IV) (7).

In Germany, BC is the most prevalent type of cancer for women (8). Consequently, the related economic burden surpasses most other cancer types (9). Inpatient care, medication, and productivity losses represent the highest proportion of the economic burden of BC (10). Total Productivity losses associated with BC were estimated at around €1,531 million, while pharmaceutical spending was €777 million in 2009 (10). Although BC costs represent a significant burden for the German healthcare system, information on the development of the healthcare costs after a cancer diagnosis is limited. The lack of information regarding BC's stage-wise direct medical costs for the German population is particularly concerning. A recent literature review (4) of the stage-wise costs of BC did not identify any analyses that considered German data. In another review of the costs of BC (11), one article evaluated German data but only estimated age-specific BC-attributable health expenditures but did not consider stage-wise costs (12).

Stage-wise treatment costs are essential to understanding the costs and benefits of new health technologies and health programs related to BC. Besides, a central assumption in any screening program is the stage shift mechanism whereby the cancer is diagnosed earlier, supposedly in less advanced stages that are may curable. Studies outside Germany suggest that early-stage cancers tend to have low healthcare costs compared to metastatic (13). The systematic review of Sun et al. (4) showed that direct medical costs are around $54,600 for stage I compared to $127,500 for stage IV. Therefore, economic evaluations of screening programs require the use of stage-specific costs rather than average costs representing all stages. Given that healthcare costs vary considerably among countries (14–17), it is necessary to establish a basis for the stage-wise costs of BC in the German context.

Only a few countries have reported the costs of BC by cancer stage (4); thus, information is limited, presumably due to cost evaluations being resource-intensive, data-scarce, and not accessible primarily related to the stage of the disease. There are two methods of estimating costs: the micro-costing approach and the top-down approach (18). The latter method typically uses macro-level healthcare expenditures. Based on these expenditures and a series of assumptions, estimating the proportion of these macro-level expenditures can be linked to a disease (e.g., using the population diagnosed with breast cancer). Depending on the assumptions, the results of a top-down aproach can vary widely.

Compared to the top-down method, the micro-costing approach is more reliable. Here costs are computed per patient in the selected sample. The collected information is summarized, and conclusions can be derived regarding average and total costs for the population. To better understand the economic burden of BC and take into account that patients can have multiple comorbidities, it is critical to determine the costs attributable to the diagnosis of BC. Ideally, costs are collected from detection to end-of-life and the estimated costs compared to those of a control group without breast cancer. Instead of a control group, and depending on data availability, the selection of only a subset of costs that are related to the particular diagnosis can be considered.

Despite being the more consistent method, micro-costing is also more resource-intensive since it requires a rich and detailed database codified (not just using the ICD 10 classification but also with clinical information) so that it is possible to determine BC attributable costs. The costs can be collected based on resource use, unit price, charges (bills), or reimbursement data. The first and the second data sources depend on the selection, follow-up, and recruitment of patients, which can be costly and usually not representative of the entire population that suffers from the disease (e.g., a sample of patients from selected hospitals or a clinical trial). If patients are selected from only one particular geographic area, results might not generalize to the entire population since care costs vary across treatment centers (specialized treatment centers vs. usual care centers) (19). The alternative is to use administrative data from Statutory Health Insurance (SHI). Depending on the population coverage, it can be representative, and, in addition, attributable costs can be estimated. However, in most countries, administrative data is not easy to access, and the use of the information is highly limited by strict confidentiality regulations, as it is in Germany. Furthermore, a critical limitation of most administrative databases is that they do not include or can not be linked to clinical information. Therefore, it is impossible to estimate stage-wise costs based on these data.

As real-world data (e.g., administrative data, clinical information for stages) is scarce, a potential alternative is the precise quantification of stage-wise BC costs *via* a modeling study. Modeling approaches make it possible to model treatment trajectories of each stage and assign costs (20). However, addressing the uncertainty around input parameters, transparency, and validation is needed to strengthen the accuracy of the model results (21). Karnon and Brown (22) suggested that a decision tree or cohort Markov model is sufficient for simple case scenarios (22). However, one should consider the model's design and feasibility regarding the available information, easy implementation, and computational time (23). While Markov models take into account the passage of time or risk over time, decision trees are well suited for transition at a point in time, very logical, linear, unidirectional, moving from left to right (without forward or backward movement) (24, 25). Hence, the decision tree has an advantage in capturing stage-wise treatment costs since every node represents a treatment option, such as treatment or no treatment.

Given the lack of data and the need to understand Germany's stage-wise costs, we aim to provide conservative estimates of the stage-wise medical costs of BC in Germany. First, we conducted a literature review of articles evaluating stage-wise costs of BC in Germany to ascertain what (if any) research has been published on this topic. Secondly, we proposed a methodology to estimate the stage-wise medical costs of BC in Germany, where we created a decision tree based on available treatment and cost information.

## 2. Methodology

### 2.1. Literature review

We conducted a rapid literature review (following the PRISMA recommendations for a systematic review with slight modifications) of the evidence available for the stage-wise cost of BC in Germany, including articles in German and English. We searched PubMed, EconLit, and Web of Science databases from 1990 to January 2020. Studies based on real-time data, such as sickness funds or patient-based costing studies, were included (See PRISMA diagram and search strategy in Supplementary material S1, Supplementary Table A1).

### 2.2. Model and costs estimation

The modeling framework was designed to establish the stage-wise cost of breast cancer. A decision tree was created and is detailed below.

#### 2.2.1. Model: Population and time horizon

The German cancer registry data provide the stage-wise incidence of BC for Germany. The data was received from the Zentrum für Krebsregisterdaten (ZfKD) Germany to design a breast cancer natural history model. We used part of the analyzed data for cost estimation of female patients aged 20 years and older diagnosed with DCIS (Ductal Carcinoma *in situ*), stage I, stage II, stage III, and stage IV for the year 2015. Furthermore, we included terminal care costs based on the stage-specific survival estimates. All those women who died due to BC were considered for end-of-life treatment (See stage-specific survival estimates in Supplementary material S2, Supplementary Table A6).

#### 2.2.2. Model: Overall approach

**Figure 1:**
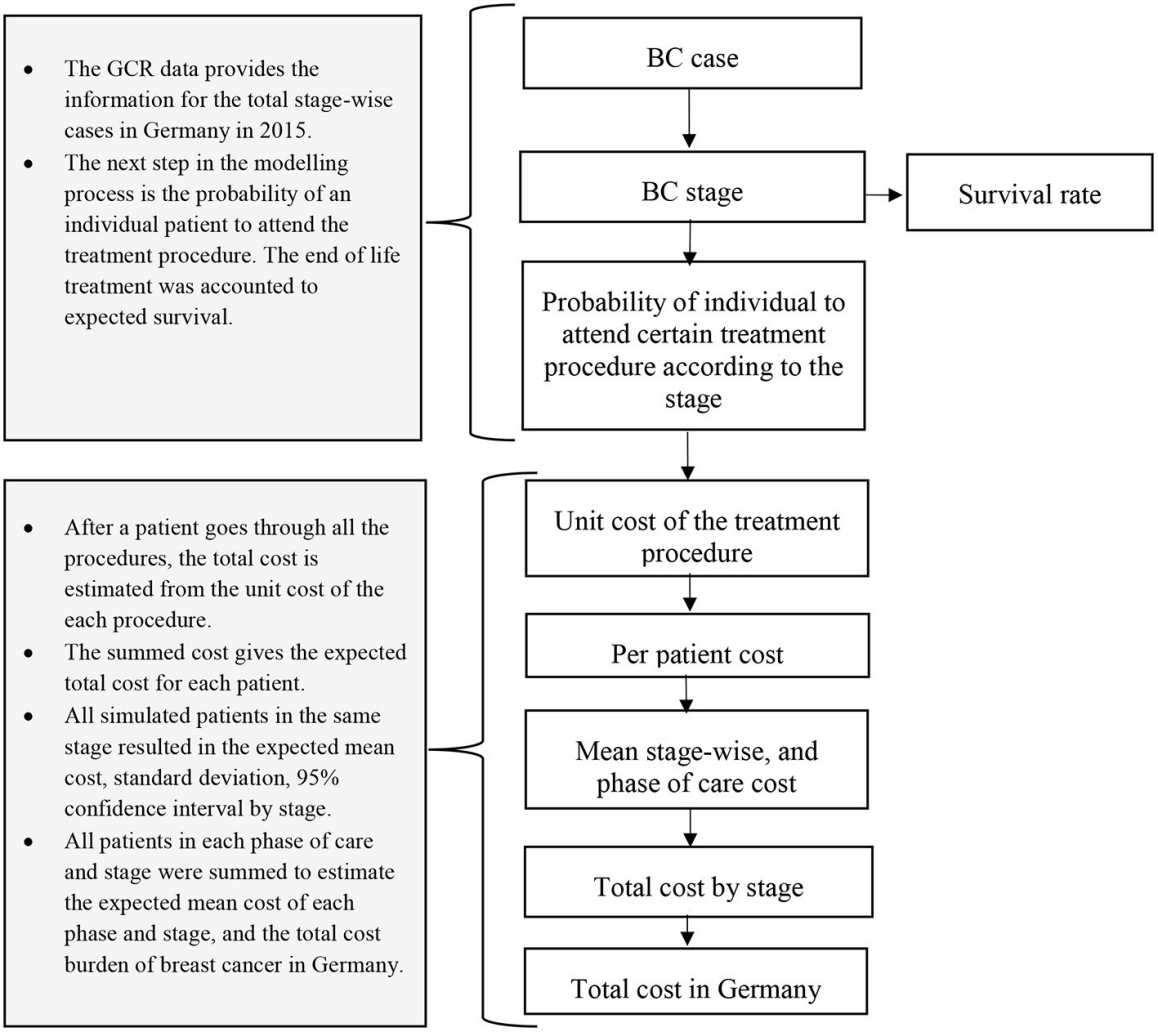
Pictorial diagram of the modeling process.

**Figure 2:**
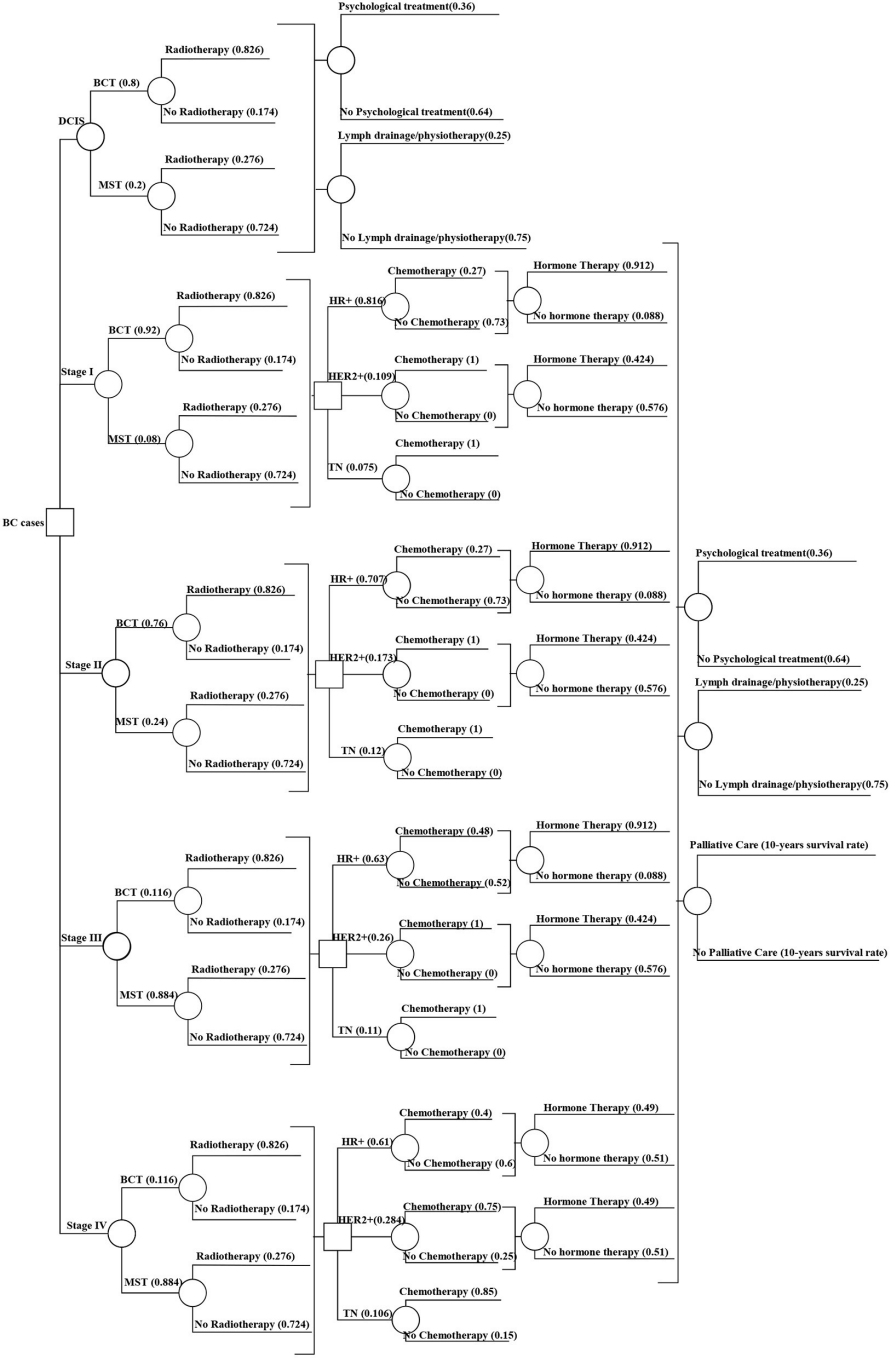
Decision tree model showing the treatment probabilities by stage. DCIS, Ductal carcinoma *in-situ*; BCT, Breast-conserving therapy; MST, Mastectomy; HR+, Hormone receptor-positive; HER2+, Human epidermal growth factor receptor 2; TN, Triple negative. Source: Authors elaboration.

This study models the trajectory of treatment and associated costs considering the number of cases in the year 2015 in Germany. Therefore, we created an incidence-based cost of illness decision tree model (Figures 1, 2) in the R programming software version 1.3.1093 (26). For each model, patient costs were estimated by multiplying the probability of getting surgery, radiotherapy, chemotherapy, hormone therapy, and end-of-life care in a particular disease stage for an associated unit cost. Additionally, the model incorporates the probability and unit cost for diagnosis, chemotherapy-associated adverse events, and psychological care. The unit cost for each care component was extracted from the existing literature.

We considered the payer's perspective and calculated direct medical costs, except for the costs of physician's visits. Additionally, direct non-medical costs were also estimated (e.g., transportation). The prices were adjusted for inflation for the year 2021 (27). Cost data were constrained to non-negative and weighted by units using gamma distribution (24).

#### 2.2.3. Input parameters

The cost of illness model has five types of inputs: (1) stage-wise annual cases of BC, (2) survival or stage-specific mortality, (3) probability of getting a specific type of treatment procedure (e.g., breast-conserving surgery or mastectomy) based on the stage at diagnosis, and (4) associated unit costs per procedure. In addition to the stage, hormone therapy and chemotherapy depend on the hormone receptor status of the patient (Hormone Receptor (HR) +ve, Human Epidermal growth factor Receptor2 (HER2) +ve or HR+ve and HER2+ve, and triple-negative). Hence, we also defined the proportion of patients eligible for getting those therapies based on their hormone receptor status (input 5).

##### 2.2.3.1. Incidence

Stage-wise cancer incidences were obtained from anonymized BC data from all the 16 German Cancer Registries available in the Center for Cancer Registry Data (ZfKD) at the Robert Koch Institute (RKI). The information provided by the RKI for BC patients covers the period from 1990 to 2015. The database includes patient-level information on sex, birth year, district, age at diagnosis, ICD10 codes C50 (invasive tumors) and D05 (DCIS), type of diagnostic confirmation, TNM, therapy, death, and date of death. This information was used to extract the stage-wise annual cases of BC (28).

##### 2.2.3.2. Diagnosis

Based on German S3 guidelines for BC (29), we assumed that all women diagnosed with BC have an indication of biopsy, and all women with positive lymph nodes, tumor size more than T2 (>2 and < 5 cm) have an indication of CT scan examination for chest and abdomen to determine the extent of disease progression staging (29). The proportion of positive lymph nodes was derived from the ZfKD's data.

##### 2.2.3.3. Treatment proportions

The ZfKD includes information about which treatments were applied to the patients: surgery, radiotherapy, hormone therapy, and/or immunotherapy. However, this information is only coded as yes or no for each treatment option. Such coding proved insufficient to estimate BC treatment proportions that mainly depend on various hormone receptor signals, tumor growth, proliferation, angiogenesis, and recurrence and are unavailable at the ZfKD. Therefore, treatment proportions were primarily informed by previously published literature. For that reason, and in addition to the review of the stage-wise costs of BC (See “Section 2.1”), we extended the search for the proportion of treatment by stage. We used the citations of the selected papers from the review. The articles that presented estimation on the proportion of stage-wise treatment were selected, and all the relevant data were extracted.

##### 2.2.3.4. Surgery

German S3 guidelines recommend breast-conserving surgery (BCS) and locoregional treatment for most early BC cases (29). It is also evident from the literature that BCS is a widely used procedure (29). For BC surgery, we used the ratio between mastectomy and BCS from a study conducted by Schrodi et al. (30) that reported BCS from Brandenburg, Dresden, Saarland, and Munich between 1999 and 2010. However, we only used the results from the most recent years, 2009–2010, which we assume reflect current practice (30). Moreover, Schrodi et al. (30) did not specify the stage Information; the authors classified surgical interventions according to primary tumor size categories (Tis, T1, T2, T3, and T4). Therefore, we used 2015 ZfKD data to compute the BC stage corresponding to the tumor size for women aged 20+. We derived proportions of stage I, stage II, stage III, and stage IV falling within each tumor size category and assigned surgical procedures accordingly. German cancer registry data shows that 90% of the T3/T4 size tumors appear in stage III and stage IV BC. BCS for T4 tumors is 11.6%; we assumed the same proportion of BCS for stage III and IV tumors appear in T1 and T2 tumor size categories and adjusted proportions of stage I and stage II accordingly (additional information on the estimations is provided in Table 1 and Supplementary material S2, Supplementary Table A5).

**Table 1:** Average per-patient stage-wise resources used and costs.

**Treatment, stage, and hormone receptor subtypes**	**Proportion of patients** **(%)**	**Treatment probabilities (%)**	**Unit cost per treatment cycle in Euro (2021 prices) (SD)**	Source^*^
**Diagnostic work-up**				
1) Diagnostic mammography	100.0	100.0	62.07	(29, 31, 32)
2) Consolatory evaluation of mammographic images	100.0	100.0	4.56	(29, 31, 32)
3) Main diagnosis				
Clarification diagnosis I: Obligatory additional imaging, facultative core biopsy using either X-ray or sonography guided	100.0	100.0	99.79	(29, 31, 32)
Supplement for vacuum biopsy	100.0	100.0	32.15	(29, 31, 32)
Histopathological evaluation of biopsy material for each of three specimens	100.0	100.0	10.79	(29, 31, 32)
Supplement for histopathological evaluation, for each of three specimens	100.0	100.0	11.79	(29, 31, 32)
Grading and immunohistochemistry (HR, HER2 neu and Ki-67)	100.0	100.0	150.85	(29, 31, 32)
**Additional diagnostic work-up for lymph node-positive & tumor size** ≧**T2**				
• CT scan (Chest and abdomen)				
**Follow up screening after removal of the primary lesion**				
1) Mammography every year				
2) Ultrasonography every year				
DCIS	0.0			(28)
Stage I	0.0			(28)
Stage II	48.0	100.0	145.73	(28, 29, 32)
Stage III	100.0	100.0	145.73	(28, 29, 32)
Stage IV	100.0	100.0	145.73	(28, 29, 32)
**Surgery**				
1) Breast-conserving surgery				
DCIS		80	4,640 (1,855)	(28, 30, 31)
Stage I		92	4,640 (1,855)	(28, 30, 31)
Stage II		76	4,640 (1,855)	(28, 30, 31)
Stage III		11.6	4,640 (1,855)	(28, 30, 31)
Stage IV		11.6	4,640 (1,855)	(28, 30, 31)
2) Mastectomy				
DCIS		20	7,045 (2,818)	(28, 30, 31)
Stage I		08	7,045 (2,818)	(28, 30, 31)
Stage II		24	7,045 (2,818)	(28, 30, 31)
Stage III		88.4	7,045 (2,818)	(28, 30, 31)
Stage IV		88.4	7,045 (2,818)	(28, 30, 31)
**Radiotherapy after surgery**				
1) Breast-conserving surgery		82.6	1,924 (769)	(31, 33)
2) Mastectomy		27.6	1,924 (769)	(31, 33)
**Hormone therapy**				
Stage I				
HR+ve	81.6	91.2	For first year: 1,181 (639) Later years: 757 (402)	(31, 34, 35)
HER 2/ neu positive	10.9	42.4		
Stage II				
HR+ve	70.7	91.2	For first year: 1,181 (639) Later years: 757 (402)	(31, 34, 35)
HER 2/ neu positive	17.3	42.4		
Stage III				
HR+ve	63.0	91.2	For first year: 1,181 (639)	
HER 2/ neu positive	26.0	42.4	Later years: 757 (402)	(31, 34, 35)
Stage IV				
HR+ve	61.0	49.0	For first year: 1,181 (639)	(31, 35)
HER 2/ neu positive	28.4	49.0	Later years: 757 (402)	(31, 35)
**Chemotherapy**				
Stage I				
HR+ve	81.6	27.0	6,846 (2,737)	(31, 35)
HER 2/ neu positive	10.9	100.0	28,517 (11,406)	(31, 35)
TN	7.5	100.0	6,846 (2,737)	(31, 35)
Stage II				
HR+ve	70.7	27.0	6,846 (2,737)	(31, 35)
HER 2/ neu positive	17.3	100.0	28,517 (11,406)	(31, 35)
TN	12.0	100.0	6,846 (2,737)	(31, 35)
Stage III				
HR+ve	63.0	48.0	6,846 (2,737)	(31, 35)
HER 2/ neu positive	26.0	100.0	28,517 (11,406)	(31, 35)
TN	11.0	100.0	6,846 (2,737)	(31, 35)
Stage IV				
HR+ve	61.0	40.0	13,111 (5,242)	(31, 35)
HER 2/ neu positive	28.4	75.0	52,414 (20,965)	(31, 35)
TN	10.6	85.0	20,592 (8,377)	(31, 35)
**Chemotherapy-induced events and treatments (only for chemotherapy-treated patients)**				
1) Neutropenic sepsis		15.0	6,213 (2,485)	(31)
2) Neulasta (pegfilgrastim)		50.0	10,586 (4,235)	(31)
3) Antiemetics		100.0	531 (212)	(31)
4) Bisphosphonates (Stage IV)		100.0	452 (181)	(31)
5) Lymph drainage/physiotherapy		25.0	1,590 (636)	(31)
6) Palliative care for patients dying of breast cancer		All women that die of BC	11,976 (4,791)	(5, 31)
7) Psychological treatment after cancer diagnosis		36.0	1,322 (528)	(31)
**Transportation**		100.0	1,776 (2,682)	(36)

HR, Hormone Receptor; HER2 neu, Human Epidermal growth factor Receptor 2; DCIS, Ductal Carcinoma *In-situ*; TN, Triple-Negative. Source: Authors' elaboration.

##### 2.2.3.5. Radiotherapy

German national guidelines recommend radiotherapy after breast-conserving surgery for local treatment of an early BC diagnosis and mastectomy and radiotherapy for advanced tumors (29). Data from Engel et al. (33) was used; they provided details on the proportion of radiotherapy for six German federal states according to the surgical procedure (See Table 1).

##### 2.2.3.6. Endocrine therapy

BC treatment decisions and the prognosis is mainly governed by the immunohistochemistry tumor markers (37). Hormone receptor-positive women receive upfront hormonal therapy (38). Hormone receptor subtypes are the combination of estrogen receptor (ER), progesterone receptors (PR), human epidermal growth factor receptor 2 (HER2), and triple-negative (TN) BC. Based on the Saarland cancer registry data, Holleczek et al. (34) estimated that 84.4% of BC cases are HR +ve, and 24.1% are HER2/neu +ve. The Munich cancer registry (39) reported 87.1% of women HR +ve and 13.46% HER2 positive. However, these studies did not report stage-wise hormone receptor status. Therefore, we used data from a Norwegian study to extract information about the stage-wise distribution of hormone receptor status (35)^.^ For the probability of getting hormone therapy for HER+ve and HER 2/ neu positive, Holleczek and Brenner (34) reported 91.2 and 42.4%, respectively, in early-stage BC, and Arnold et al. (31) reported 49% of HR +ve or/and HER2+ve women were treated with endocrine therapy for metastatic BC (See Table 1).

##### 2.2.3.7. Chemotherapy

Chemotherapy is preferred in triple-negative and HER2/neu-positive BC cases (29, 31). Therefore, we assumed a 100% probability of getting chemotherapy in those groups. Estrogen and/or progesterone receptor-positive women having grade 3 or 4 tumors are also recommended for chemotherapy. We used proportions estimated by Arnold et al. (31) for chemotherapy and associated adverse events.

##### 2.2.3.8. Palliative care and end-of-life treatment

Cancer patients in advanced stages have a higher risk of spending most of their end-of-life days in healthcare settings. Studies have demonstrated that intensive medical care has increased in the last phase of life (40). Based on the Munich cancer registry reported BC survival data, we modeled each woman's stage-wise probability of death. All women dying of BC were considered to have received palliative care (See stage-specific survival estimates in Supplementary material S2, Supplementary Table A6).

##### 2.2.3.9. Mental health

A cancer diagnosis has profound mental health effects (41). Patients treated with BC also experience psychological consequences and disturbances in their ordinary lives. Therefore, psychological therapy was also included as a potential treatment in the model from the literature (31).

#### 2.2.4. Unit costs and non-medical costs

For the diagnostic procedure paid by SHI in Germany, a unit cost was assigned from the price catalog of the Kassenärztliche Bundesvereinigung (KBV) (32). Treatment costs were assigned from the published literature (31, 32, 42, 43). Additionally, the analysis includes non-medical costs in terms of travel expenses. Recent estimations by Kreis et al. (36) on travel costs borne by BC patients were extracted; these are based on data from the insurance company Allgemeine Ortskrankenkasse (AOK).

#### 2.2.5. Outputs

Per patient, stage-wise BC costs (i.e., DCIS, stages II, III, IV, and I) were estimated. Costs are also provided by the initial, intermediate, and terminal phases of care and overall cost per incident case. The overall cost of BC for 2015 was estimated compared to the results with the cost of illness data provided by the Federal Statistics Office (2). The estimates are reported as a mean cost per patient in Euros for the year 2021.

#### 2.2.6. Validation

The lack of empirical evidence on stage-wise cost in Germany makes it almost unfeasible to validate the model results using external data. However, for the German BC patients, the current literature elucidates only costs per incident case for the initial phase of care (first 11 months), terminal phase of care (last 11 months of life), and the intermediate phase (any period of that is not classified in the initial or the terminal phases) (36). Consequently, the model outputs for stage-wise costs cannot be validated; therefore, we used the estimation by phases (i.e., the initial, intermediate, and terminal phases) presented by Kreis et al. (36) and adjusted the estimates for inflation to the year 2021.

In order to compare the model outputs and the estimates provided by Kreis et al. (36), we assigned costs to BC patients according to the disease management phases: (1) all simulated women in the model that do not die of BC were considered for the initial phase of care. This phase comprises all components except hormonal therapy (extended for 2–5 years) and surveillance; (2) the intermediate phase comprises the surveillance and hormone therapy cost; and (3) the terminal phase consists of all the women that die of BC.

#### 2.2.7. Sensitivity analysis

The uncertainty surrounding the stage-wise cost estimates stems from uncertainty around the proportion of treatments and unit costs. Therefore, we conducted deterministic sensitivity analysis by varying treatment proportions to zero for all the procedures (lower case scenario) and 100% (upper case scenario) except for surgery and survival rate. The breast-conserving surgery was considered for all BC patients in the lower case scenario, and mastectomy was considered for all BC patients (upper case scenario); for end-of-life treatment, a 5-year survival rate was considered for the lower and a 15-years survival rate for the upper case scenario.

Regarding the cost parameters, we varied ±20 percentage of the mean unit cost for breast-conserving surgery, mastectomy, chemotherapy, psychological therapy, radiotherapy, hormone therapy, and palliative care.

## 3. Results

### 3.1. Systematic review

Our search identified 343 titles, out of which 57 were duplicates. According to the defined inclusion and exclusion criteria, 244 articles were excluded after reviewing the title and abstract and 35 articles after reading the full-text document (See PRISMA diagram; inclusion and exclusion criteria are listed in Supplementary material S1.1, Supplementary Figure A1).

Seven articles were costing studies evaluating BC costs in the German context. Out of the seven, five studies (43–47) used case scenarios based on German S3 BC treatment guidelines and other sources from the literature, and costs were calculated mainly using predefined national tariffs such as the DRG (diagnosis-related groups) for inpatient care, the EBM (German Uniform Evaluation Standard) for outpatient care and the Rote Liste for drug prices (43–47). These studies reported a BC cost per patient; however, the estimates cannot be linked to the disease stages. Additional information on these five studies based on case scenarios is presented in Supplementary material S1.2.1, Supplementary Tables A.2, A.3.

Additionally, out of the seven studies, two (12, 36) used real-time data from SHI (sickness funds) to estimate BC treatment costs from the perspective of the German healthcare system and the payers. These studies used a bottom-up approach for costing. Gruber et al. (12) estimated the BC costs for 1990 and adjusted them for 2010 inflation. They used data from four sickness funds aggregated into two datasets and found that the average annual cost per woman ranges from €6,000 to €10,000. The cost was higher for younger women (about €9,000) than for older (about €3000). Kreis et al. (36) used 2011–2014 data from AOK statutory health insurance. They calculated an initial phase incremental cost of €21,498, an intermediate phase cost of €2,620, and a terminal phase cost of €34,513 per incident case. Even though these two studies are based on large sample sizes, limitations hinder the usage of the information. Gruber et al. (12) results are based on over two decades old cost data. Since 1999, significant changes have occurred: technological advancement in BC diagnosis and treatment, population dynamics, the introduction of the BC screening program in 2005, and the DRG flat rate payments in 2004. Therefore, Gruber et al. (12) estimates may no longer reflect today's costs. Kreis et al. (36) present more recent approximations on the costs of BC, with the advantage of providing estimations by phase of care. However, a significant limitation in both studies is the lack of information about stage-wise costs.

Overall, the rapid review suggests a lack of information about the stage-wise costs of BC in Germany since we could not find a single relevant study that provides an estimation. Additionally, the review indicates that sickness funds collect their data with a strong emphasis on the German ICD-10 classification, which cannot be translated to stage-wise cost without additional pertinent clinical information such as tumor size at detection and lymph node involvement.

### 3.2. Estimates of the medical cost of BC in Germany

Our study cohort included 75,942 patients diagnosed with BC in 2015. Based on the ZfKD data, the stages are distributed as follows: 6,050 cases of DCIS, 27,866 of stage I, 27,571 of stage II, 8,692 of stage III, and 5,763 of stage IV (See Supplementary material S2, Supplementary Table A4).

**Figure 3:**
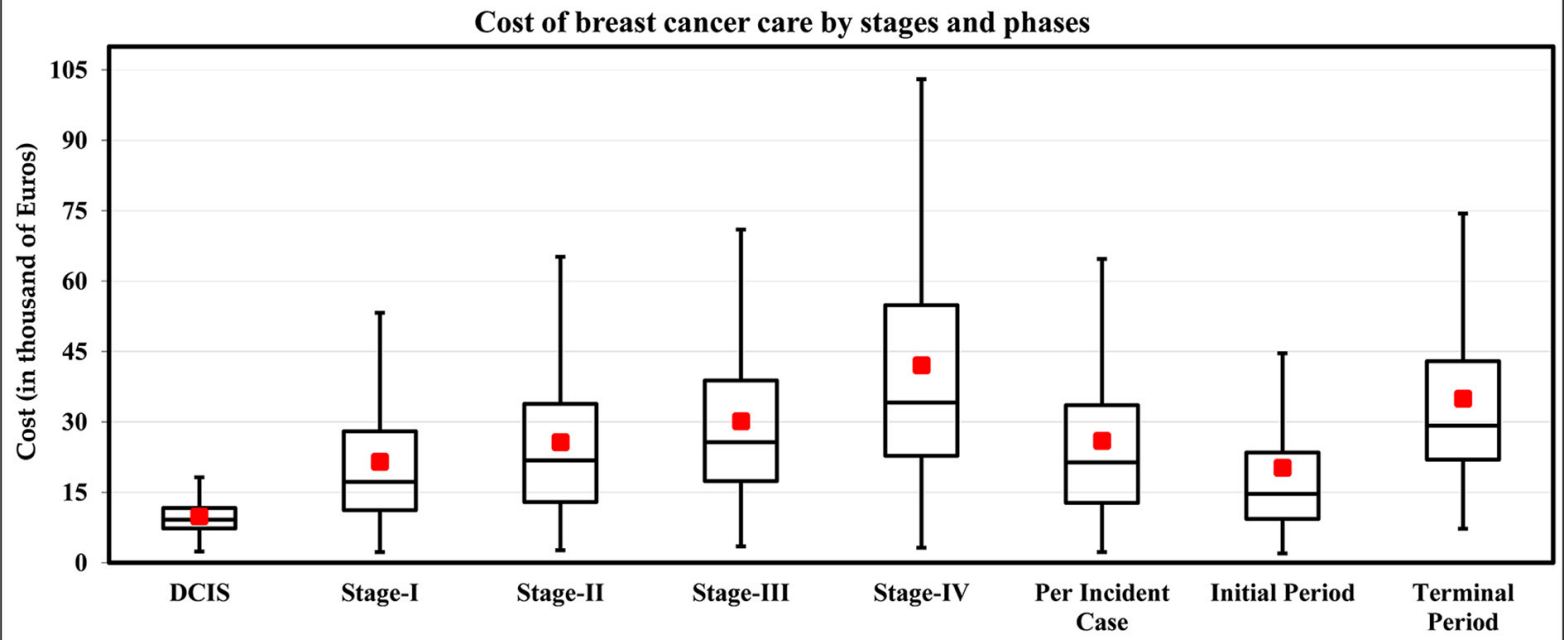
Estimated stage-wise medical cost of BC per patient in Germany for the year 2015 (in Euros, 2021 prices). Box and whisker plots showing the median (black line), mean (red square box), minimum and maximum values (whisker), and first and third quartiles (box). Note. The cost of the intermediate phase is too low (mean = €851, SD = €481) for the graph. Source: Authors' elaboration.

Based on the seven articles identified in the literature review, German guidelines, and the ZfKD data, the proportions for patients and treatments by stage were selected and are presented in Table 1. The estimated cost per incident case was €25,932 per patient, and the average expected cost of invasive BC per patient during the initial and terminal care was €20,284 and €34,963, respectively. The results are presented in Table 2 and Figure 3. As expected, the costs increased with the stage of the disease, where patients in Stage IV had the highest costs. The high costs estimated for metastatic BC patients are due to the impact of end-of-life treatment costs. The treatment costs for late-stage BC (Stage III and Stage IV) are 53% higher than early BC (stage I and II), Stage II is 19.3%, stage III is 40.1%, and stage IV is 95% higher than the costs in stage I.

**Table 2:** Model estimated mean medical costs of BC in the Germany for the year 2015 (in Euros, 2021 prices) and validation from the German literature.

	**Number of cases**	**Mean [SD]**	**CI LL**	**CI UL**	**Overall cost**	**Validation** **Kreis et al. (36)**	**Muller et al. (43)**	**Schrauder et al. (47)**
***DCIS***	6,050	€9,838 [€3,755]	€9,744	€9,933	€59,520,835			
***Invasive***								
Per-incident case		€25,932 [€17,486]	€25,801	€26,061	€1.812 billion			
Initial phase^*^		€20,284 [€14,779]	€20,115	€20,382		€21,499^*^		
Intermediate phase		€851 [€481]	€848	€855		€2,619		
Terminal phase		€34,963 [€18,691]	€34,721	€35,205		€34,513		
Stage I	27,866	€21,523 [€13,917]	€21,359	€21,686			€20,000^1^	€20,394^2^
Stage II	27,571	€25,679 [€16,097]	€25,489	€25,869				
Stage III	8,692	€30,156 [€17,289]	€29,793	€30,520				
Stage IV	5,763	€42,086 [€26,443]	€41,403	€42,769			€45,000^1^	€39,029^2^

CI LL, Confidence interval lower limit; CI UL, Confidence interval upper limit; DCIS, Ductal Carcinoma *In Situ*.

^*^First 11 months, without end-of-life-care. ^1^The study mentioned the direct medical cost for early and metastatic BC only (not all stages) using the treatment proportions from the real time data from the German Consortium for Hereditary Breast and Ovarian Cancer. ^2^The study mentioned the direct medical cost for early and metastatic BC only (not all stages). Source: Authors' elaboration.

A significant increase in the cost between stages was observed during the initial phase of care (curative phase, first 11 months). On average, the model estimated the average expected costs are 17.8, 36.5, and 95.9% higher for stages II, III, and IV compared to stage I. The same pattern was observed in the terminal phase of care between stages I through IV. The cost drivers are mainly surgery and chemotherapy, contributing 61% of the total cost of care, while palliative care contributed 15% of the total cost of care.

The model estimated the total medical cost of BC in Germany is €1.824 billion for incident cases in 2015. The model estimated value is lower than that provided by the Federal Statistics Office, which reported BC cost of illness of around €2.15 billion in 2015 (€2.34 billion, adjusted to 2021) for the female population (See Supplementary material S3, Supplementary Table A7).

### 3.3. Validation

We externally validated our results with those in the German literature, Kreis et al. (36) (Table 2), which used the large AOK statutory health insurance database. For the initial and terminal phases, our results were 6.8% lower and 1.3% higher, respectively, than those from Kreis et al. (36). These findings suggest that our estimations are comparable with those of real-time data. The model costs for the intermediate phase are not consistent with the German estimates. We also compared the results of the published German literature that mentioned the cost of early and metastatic BC (43, 47) (Table 2), and our estimates are consistent.

### 3.4. Sensitivity analysis

Supplementary material S4, Supplementary Figure A2 consists of tornado graphs showing the impact of different input parameters on cost estimates. For the follow-up period of BC patients and the proportion of patients receiving chemotherapy, we observed that the overall and mean stage-wise cost of BC vary substantially in low and higher case scenarios. The follow-up period of BC patients up to 15 years in the model translates to a higher number of women dying due to BC, impacting the mean cost of care in the early stages compared to the late stages. If chemotherapy is not offered to all patients, irrespective of the stage, the mean costs of stage I, stage II, and stage III decrease by approximately 10%, and in stage IV, the costs decrease by 50%. Additionally, submitting all patients diagnosed in the early stage to BCS has little impact on the mean cost of care, while offering mastectomy substantially increases the mean cost of care in the early stages.

## 4. Discussion

To the best of our knowledge, the present study is the first to estimate the stage-wise medical costs of BC considering the German context, including a review of current literature. The review indicated an inherent limitation in collecting sickness funds data, which hinders the estimation of stage-wise costs. The data is collected for reimbursement purposes; therefore, diagnosis is mentioned based on the German ICD-10 classification. Thus, claims data lack the clinical information for tumor size and lymph-node involvement necessary to understand BC costs by disease stage (4). Furthermore, the patient-level primary data collection to estimate stage-wise cost is resource-intensive and not feasible across the country. To fill this gap, we build a decision tree model that combines information from the RKI, clinical guidelines, published literature, and unit costs from the KBV. We estimated that the mean BC medical costs were €21,845 in stage I, €25,753 in stage II, €29,825 in stage III, and €42,796 in stage IV. We also report estimations regarding the average cost per patient (€26,107) and per phase (initial €20,384 and terminal €35,125). Although the model estimates for the costs of BC are close to those previously published for the German context (36, 43, 47), this does not necessarily guarantee that the stage-wise medical costs estimated reflect the actual costs. Because of the heterogeneity in the model inputs, actual costs cannot be assessed accurately. Micro-costing studies remain a more reliable method to estimate costs. Since SHI data are collected for non-scientific purposes, especially billing, they are frequently used for costing studies. Therefore, in order to get stage-wise cost, it is highly recommended that SHI data should be linked with German cancer registry data. However, the data linkage approval process is challenging in Germany due to stringent data protection laws and the engagement of several authorities, making the approval process challenging. An alternative would be collecting clinical data (stage at diagnosis) by the SHI agencies so that cost information can be linked to the BC stages resulting in stage-wise BC costs.

Nevertheless, compared with European studies (15, 17, 48), the model estimated stage-wise cost aligns with the Belgium study (48) conducted in 2015 assessing direct medical costs. The study estimated the mean cost of DCIS, stages I, II, and III to be €9,063, €17,624, €25,209, and €30,985 (2021 prices). However, the data in this study (48) was limited to one hospital and 107 patients (only two patients in stage IV). On the contrary, we observed a higher mean stage-wise cost in our estimations compared to Italian (15) and Portuguese studies (17, 49). That could be mainly because these studies did not explicitly include end-of-life care and have a shorter follow-up period that potentially underestimates the cost of end-of-life care. However, their results are consistent with our results regarding the percentage increase in advanced-stage cancers compared to stage I costs (see Table 3). Given the heterogeneity in cost structures and the epidemiological factors between and to some extent within countries, BC treatment cost estimates are significantly variable, and their transferability remains questionable.

**Table 3:** Models estimated mean direct medical costs (including percentage change compared to Stage I), Euros 2021.

	**Model estimated Mean cost - Germany** **(% increase compared to Stage I)**	Lemhouer et al. (48) – Belgium (% increase compared to Stage I)^*^	Sun L. et al (4) – Systematic review (% increase compared to Stage I)^**^	**Capri and Russo (15) - Italy (% increase compared to Stage I)**	**Harfouche et al. (17) - Portugal** **(% increase compared to Stage I)**	**Brandao et al. (49) - Portugal** **(% increase compared to Stage I)**
DCIS	€9,838	€9,063	€9,744		€5,429	
Stage I	€21,523	€17,624	€24,586	€9,361	€8,586	€9,796
Stage II	€25,679 (19.3%)	€25,209 (43%)	€32,525 (32%)	€11,169 (19%)	€13,786 (60%)	€13,839 (41%)
Stage III	€30,156 (40.1%)	€30,985 (76%)	€47,831 (95%)	€13,952 (49%)	€16,755 (95%)	€15,957 (63%)
Stage IV	€42,086 (95.5%)	€7,581^*^	€51,372 (109%)	€13,318 (42%)	€20,478 (138%)	-

DCIS, Ductal Carcinoma *In Situ*. ^*^only two patients in Stage IV for cost estimation ^**^The cumulative mean of all studies included in the systematic review (nine studies from US, four from European Union, and eight from other countries world-wide). Source: Authors' elaboration.

The BC treatment costs are higher in advanced stages, suggesting that screening detection and prevention strategies are central to reducing costs. This study adds substantial evidence to the current literature using reproducible and robust methods, allowing model adjustments for new treatments, mortality rates, disease recurrence, and changes in treatment guidelines (20). Given the recent advancements in personalized oncology, newer diagnostic tools, and targeted medicine, the average and stage-wise cost of care could substantially increase. Therefore, modeling studies may be instrumental in projecting the national medical cost of breast cancer. However, model adjustments and the inclusion of new treatments, recurrent and progressive BC are contingent upon the availability of data.

Results in our study are susceptible to assumptions regarding the follow-up period. Even if advanced cancer offers poor survival rates, thus assuming a short time horizon having little impact on the mean cost of BC in advanced stages, for early-stage cancers, longer follow-ups are needed to accurately estimate the number of women dying due to BC. That is paramount for estimating end-of-life care costs. Moreover, factoring age and BC survival in the cost model are essential. It is estimated that the overall cost among younger cancer patients is 20% to 50% higher compared to all patients for the initial and terminal phases of care (50). The higher costs may be attributed to the aggressive BC care in young women because they are more likely to be diagnosed with BC stages II and III (stage 2 BC: 44.3 vs. 29.9%, respectively; stage 3 BC: 14.0 vs. 7.7%, respectively), triple-negative BC (21.2 vs. 13.8%, respectively), and HER2-positive (HER2+: 26.0 vs. 18.6%, respectively) compared to middle-aged women (50, 51). Therefore, future studies should consider age while estimating stage-wise costs.

We recognize that the modeling study is subject to limitations. First, the model only accounts for incident cases. Therefore, the cost of surveillance, mainly governed by prevalent BC (314, 546; 5-years prevalence in 2015) (8) cases, is underestimated. For example, the model estimates of the intermediate phase (€851) are inconsistent with Kreis et al. (36) estimates of €2,528. Also, the model estimate of €1.81 billion in medical costs of BC incident cases for 2015 is not in line with the overall economic burden (€2.15 billion in costs for female BC cases in Germany in 2015) (2). Second, the German cancer registry does not provide information on BC progression, recurrence, or associated treatment pathways. This resulted in the model being unable to account for recurrence and progression costs. Consequently, the costs associated with intermediate phase and stages were underestimated. Third, the modeling study relied on multiple data sources to estimate stage-wise costs, and the approach is prone to uncertainties and variability. Fourth, a modeling study could not reflect the heterogeneity and complexity of the management of BC in different health settings (e.g., specialized BC treatment centers vs. usual healthcare settings). Nonetheless, in the absence of documented clinical information needed to estimate stage-wise costs, these modeling methods could offer feasible estimates of the cost of care.

## 5. Conclusion

The literature review did not identify any study published in Germany for stage-wise costs of BC. This reflects fundamental shortfalls in the German data collection methods and data linkage between SHI claims data and the German cancer registry data, leading to a major hurdle in calculating the stage-wise cost. This affects cost estimations for not only BC but also all types of cancer. Therefore, we cannot estimate the reduction in the cost of care due to early detection by stage shift and the cost-effectiveness of screening programs.

Until the German data adequately address the lack of information regarding stages and associated costs. The conservative estimates from the model presented here can be used for future economic evaluations. Considering the increasing cost burden of cancers, and the need of efficient cancer screening programs, our study underscores the importance of understanding the importance stage-wise costs of BC mainly from the economic evaluation perspective. As the population ages, future analyses should focus on the impact of prevalent cases on the BC and palliative care costs.

## Data availability statement

The original contributions presented in the study are included in the article/Supplementary material, further inquiries can be directed to the corresponding authors.

## Author contributions

SK: conceptualization, literature search, data analysis, model development, visualization, and writing—review and editing. KH-V: conceptualization, visualization, and writing—draft and editing. DH: literature search and writing—review. MS: supervision, writing, and editing. All authors contributed to the article and approved the submitted version.
