# Supplementary material for: Estimation of the stage-wise costs of breast cancer in Germany using a modeling approach
Source: Front Public Health. 2023 Jan 6;10:946544. doi: 10.3389/fpubh.2022.946544 (PMC9853539; doi:10.3389/fpubh.2022.946544)
Supplement: Supplementary file 1 [file Table_1.docx]

**Estimation of the stage-wise costs of breast cancer in Germany using a modelling technique.**

**Supplementary material**

Shah Alam Khan^1,2*^, Karla Hernandez-Villafuerte^1^, Diego Hernandez Carreno^1^ , Michael Schlander ^1,2,3^

^1^Division of Health Economics, German Cancer Research Center (DKFZ), Heidelberg, Germany

^2^Medical Faculty Mannheim, University of Heidelberg, Mannheim, Germany

^3^Alfred Weber Institute (AWI), Faculty of Economics & Social Sciences, University of Heidelberg, Heidelberg, Germany

*** Correspondence:**Shah Alam Khan
[shahalam.khan@nct-heidelberg.de](mailto:shahalam.khan@nct-heidelberg.de)

dr.alamsolangi@gmail.com

**S1 – Rapid Review**

**S1.1 Search Strategy**

We conducted a rapid review of the cost of breast cancer in Germany. This process differs from systematic literature reviews in that only one researcher (SK) screened and evaluated all articles. Three databases were searched (PubMed, Web of Science & Econ Lit).

The inclusion and exclusion criteria are detailed in Table A.1, and the details of article selection are provided in Figure A.1.

***Table A.1. Inclusion and Exclusion Criteria based on PRISMA Guidelines***

| **PICOS** | **Include** | **Exclude** |
| --- | --- | --- |
| Population | - German female breast cancer patients. | - Studies focused on the male breast cancer only. |
| Intervention | - German female breast cancer patients. | - Cost not calculated in patient level data - Cost using treatment pathways - Review articles |
| Comparator | - No restrictions |  |
| Outcome & Studies | - Stage-wise cost of breast cancer - Costing studies using primary data | - No treatment cost by stage |
| Time | 1st January 1990 to January 2020 |  |
| Setting & Country | - Germany |  |

Source: Authors elaboration according with PRISMA guidelines(1)

Duplicates removed (n = 57)

Full-text articles assessed for eligibility
(n = 42)

Costing studies selected for data extraction
(n = 7)

Records identified through database searching

(n = 343)

Studies reporting stage-wise costs of BC using real data (n = 0)

Records excluded after title and abstract screening

(n = 244)

Records screened
(n = 286)

- Reported hypothetical case scenarios (n = 5)
- Real time data but no information about stage-wise cost

(n = 2)

Records excluded after full read (n = 35)

***Figure A.1. PRISMA Flow Diagram - Study Selection***

**S1.2 Included studies**

### S1.2.1 Cost calculation based on case scenarios

Five studies used flat rates from the DRG (Diagnosis-related group), KBV (Kassenärztliche Bundesvereinigung), and the Rote Liste, cost estimates for different hypothetical case scenarios. The scenario presented by Hammada et al.(2) considered Stage IIb and calculated costs for three scenarios: the high-cost scenario in a treatment cost equal to €53,957, the base cost of €25,374 and low-cost scenario of €23,337. Schrauder et al.(3) assumed a scenario for hormone receptor-positive (HR+ve) postmenopausal women and reported a one-year treatment cost of €18,361.62 for early BC, €15,044 for recurrent BC, and €39,028 for metatstic BC. Muller et al.(4) considered 60% of women to be triple-negative, 10% human epidermal growth factor receptor (HER2+ve), and 30% HR+ve. They estimated €20,000 costs for early BC and €45,000 for metastatic. Blank et al.(5) reported €31,699 average per patient treatment costs for estrogen receptor-positive and human epidermal growth factor negative women. Further details are given in Table A.2 and A.3.

**Table A.2. Costing studies evaluating BC costs in the German context.**

| **Study** | **Method** | **Perspective** | **Data** | |
| --- | --- | --- | --- | --- |
|  |  |  | **Representivity** | **Source** |
| Lux et al(6). 2011 | Bottom-Up Approach | Healthcare sector | ***Case scenario***: Post menopausal & HR+ve¹ | German treatment guidelines |
| Gruber et al(7).  2012 | Bottom-Up Approach | Payers Perspective | 25.1 million  (57% of German female population) | Sickness Funds data |
| Hamada et al(2). 2013 | Bottom-Up Approach | Healthcare sector | ***Case scenario:***  Stage IIb  Low cost: Triple –ve  Base case: HR+ve  High cost: Triple +ve | German treatment guidelines |
| Blank et al.  2015(5) | Bottom-Up Approach | Healthcare sector | ***Case scenario:*** ER+ve², HER2-ve³ BC | German treatment guidelines |
| Schrauder et al(3). 2017 | Bottom-Up Approach | Healthcare sector | ***Case scenario:*** Post menopausal & HR+ve | German treatment guidelines |
| Muller et al(4).  2018 | Bottom-Up Approach | Payers Perspective | ***Case scenario:*** 60% of women were assumed to be triple-negative, 10% HER2+ve and 30% HR+ve | German Consortium for Hereditary Breast and Ovarian Cancer |
| Kreis, Plöthner et al(8). 2020 | Bottom-Up Approach | Payers Perspective | AOK statutory health insurance data: 4.3 million German women | Sickness Funds data |

HR+ve = Hormone receptor positive, HER2+ve = Human epidermal growth factor receptor 2
Source: Authors’ elaboration.

DRG-related costs and payments are fixed payments based on patients’ principal diagnoses and treatment procedures conducted. The accuracy of the studies that use national tariff system to estimate average costs per patient is under question. It might not represent the cost varibiality with patients of same diagnosis; therefore, cost of illness studies that reflect actual transactions should be used(9). For instance, from the ductal carcinoma in situ (DCIS) patients, around 80% had breast conservation surgery which cost €4,391 (±€1,756), while 20% had a complete mastectomy which cost €6,667 (±€2,667)(10). Similarly, Heerey et al.(11) argue that, in pharmacoeconomic evaluations, DRGs based cost estimations may not represent the actual resources used since there are variations in utilization of medical services, which depends on disease characteristics, length of stay and choice of treatment. In this regard, case scenarios do not consider individual patient variation in the length of stay, which is a critical factor in cost determinations. Consequently, the cost calculated in these studies did not explicitly describe the actual treatment costs.

***Table A.3***: ***The cost components and the total cost of breast treatment***

| **Study** | **Cost component** | | | | | | | | | **Source** | **Results** |
| --- | --- | --- | --- | --- | --- | --- | --- | --- | --- | --- | --- |
|  | **Direct Cost** | | | | | | | | **Indirect cost** |  |  |
|  | **Medical Cost** | | | | | | | **Non-medical Cost** |  |  |  |
|  | **Inpatient** | | | | | | **Outpatient** |  |  |  |  |
|  |  | **Treatment** | | | | |  |  |  |  |  |
|  | **Diagnosis** | **Surgery** | **Chemotherapy** | **Radiotherapy** | **Drug** | **Other** |  |  |  |  |  |
| Lux et al(6).  2011 |  |  |  |  |  | Adverse events & dual-energy X-ray absorptiometry |  |  |  | DRG, KBV, Rote Liste | Contralateral BC³: €18,361.62  Recurrent BC: €15,044  Metastatic BC: €19,514.27 |
| Gruber et al(7).  2012 | Hospital costs (physician costs, nursing care, cost of hospital stay ) | | | |  | Sickness benefit |  |  |  | Sickness Funds data | BC attributable cost  €3,000 to €9,000 |
| Hamada et al(2).  2013 |  |  |  |  |  | Follow up care |  |  |  | DRG, KBV, Rote Liste | Base case: €25,374  Low cost: €23,337  High cost: €53,957 |
| Blank et al.  2015(5) |  |  |  |  |  | Adverse events, end of life treatment (last 4 weeks) |  |  |  | DRG, KBV, Rote Liste | Average per patient cost: €31,699 |
| Schrauder et al(3).  2017 |  |  |  |  |  | Follow up care |  |  |  | DRG, KBV, Rote Liste | Early BC: €18,361.62/ 1year &  €20,393.74/ 5 year  Recurrent BC: €15,044/ 1 year & €17,076.13/ 5 years  Metastatic BC: €39,028.54 |
| Muller et al(4).  2018 |  |  |  |  |  | Adverse Events |  |  |  | GCHBOC, DRG, KBV, Rote Liste | Early BC €20,000  Metastatic BC €45,000 |
| Kreis, Plöthner et al(8). 2020 |  |  |  |  |  | Sickness benefit,  Remedies/medical aids, and rehabilitation |  |  |  | Sickness Funds data | **With sickness benefits**  Overlall cost (by phase):  First 11 months (initial)¹: €26,227.  Intermediate²: €6,615  Terminal²: €39,044  BC-attributable cost (by phase):  First 11 months (initial)²: €23,084  Intermediate ²: €3,067  Terminal ²: €35,761  **Without sickness benefits**  Overall cost (by phase):  First 11 months (initial)²: €24,224  Intermediate²: €6,013  Terminal²: €37,276  BC attributable cost (by phase):  First 11 months (initial)²: €21,216  Intermediate²: €2,585  Terminal²: €34,059 |

DRG= Diagnosis-related group, KBV= Kassenärztliche Bundesvereinigung

¹ This cost estimates from this particular paper are presented in a detail because we used these estimates to compare with our model outputs
²The given estimates of the cost are inflated to 2021
Source: Authors’ elaboration**.**

**S2 – Model Input Parameters**

**German Cancer Registry Data**

The cancer registry data for breast cancer are being evaluated for model inputs. The BC registry data provides information about the incident cases from 1999 to 2015. We chose to analyse the years 2015 because the Federal statistical office Germany also published cost of illness of breast cancer, and a recent publication for the incident cost of breast cancer used current AOK data.

The cancer registry reported 69,892 cases in 2015. However, 34.8% of the reported cases lack information for size and lymph node involvement. Therefore, we used MICE imputation method in R statistical software to impute the missing information.

The stages specific cases were further analysed to compute breast cancer stages corresponding to the size of the of the tumour. The details of the database are given in the following table A.4 and A.5:

***Table A.4. Age-wise stage specific number of cases, and incident rate of BC per 100,000 women in 2015 in Germany.***

| Age | Cases (invasive) | Age-wise incidence rate | DCIS incidence rate | Stage I incidence rate | Stage II incidence rate | Stage III incidence rate | Stage IV incidence rate |
| --- | --- | --- | --- | --- | --- | --- | --- |
| 20 - 24 | 40 | 1.96 | 0.14 | 0.55 | 1.00 | 0.18 | 0.09 |
| 25 - 29 | 286 | 11.60 | 0.58 | 3.59 | 5.14 | 1.43 | 0.89 |
| 30 - 34 | 827 | 34.79 | 1.98 | 11.78 | 15.59 | 3.89 | 1.55 |
| 35 - 39 | 1555 | 67.42 | 3.80 | 22.38 | 30.27 | 7.45 | 3.52 |
| 40 - 44 | 3077 | 132.93 | 8.46 | 47.47 | 55.84 | 14.81 | 6.35 |
| 45 - 49 | 5981 | 199.83 | 14.09 | 72.43 | 83.42 | 19.68 | 10.21 |
| 50 - 54 | 8452 | 287.49 | 42.10 | 120.65 | 90.86 | 22.47 | 11.41 |
| 55 - 59 | 7164 | 267.27 | 30.47 | 115.63 | 83.74 | 24.06 | 13.38 |
| 60 - 64 | 8118 | 340.80 | 37.00 | 153.25 | 105.10 | 27.72 | 17.73 |
| 65 - 69 | 7943 | 392.39 | 39.57 | 179.03 | 117.21 | 32.38 | 24.21 |
| 70 - 74 | 7071 | 351.91 | 18.66 | 121.83 | 135.40 | 43.92 | 32.10 |
| 75 - 79 | 8492 | 371.35 | 14.58 | 110.49 | 151.50 | 54.66 | 40.12 |
| 80 - 84 | 5267 | 359.62 | 9.77 | 83.99 | 152.43 | 65.32 | 48.11 |
| 85 – 90 | 3516 | 364.40 | 6.41 | 72.29 | 154.25 | 72.39 | 59.05 |
| 90+ | 2103 | 382.38 | 3.61 | 79.54 | 147.36 | 85.13 | 66.74 |
| Stage I | 27,866 |  | | | | | |
| Stage II | 27,571 |  |  |  |  |  |  |
| Stage III | 8,692 |  |  |  |  |  |  |
| Stage IV | 5,763 |  |  |  |  |  |  |

DCIS = Ductal carcinoma in-situ

***Table A.5. Proportion of breast conserving surgery and mastectomy for each stage according to the category of primary tumor size (T1 to T4)***

| Size category T1 | | | | | | |
| --- | --- | --- | --- | --- | --- | --- |
| Stage | Size | Number of cases | Proportion of treatment | BCS | All T1 cases assigned BCS | Schrodi et al. ^(12)^ |
| 1 | 1 | 27866 | 0.92 | 25640 |  |  |
| 2 | 1 | 5532 | 0.8 | 4426 |  |  |
| 3 | 1 | 1000 | 0.116 | 116 |  |  |
| 4 | 1 | 691 | 0.116 | 80 |  |  |
|  | | 35093 |  | 30262 | 0.86 | 0.86 |
| Size category T2 | | | | | | |
| Stage | Size | Number of cases | Proportion of treatment | BCS | All T2 cases assigned BCS | Schrodi et al. ^(12)^ |
| 1 | 2 | 0 |  | 0 |  |  |
| 2 | 2 | 20891 | 0.77 | 16086 |  |  |
| 3 | 2 | 2709 | 0.116 | 314 |  |  |
| 4 | 2 | 1873 | 0.116 | 217 |  |  |
|  |  | 25473 |  | 16617 | 0.652 | 0.65 |
| Size category T3 | | | | | | |
| Stage | Size | Number of cases | Proportion of treatment | BCS | All T2 cases assigned BCS | Schrodi et al. ^(12)^ |
| 1 | 3 | 0 |  |  |  |  |
| 2 | 3 | 1151 | 0.32 | 368 |  |  |
| 3 | 3 | 2291 | 0.116 | 266 |  |  |
| 4 | 3 | 875 | 0.116 | 101 |  |  |
|  |  | 4317 |  | 735 | 0.17 | 0.172 |
| Size category T4 | | | | | | |
| Stage | Size | Number of cases | Proportion of treatment | BCS | All T4 cases assigned BCS | Schrodi et al. ^(12)^ |
| 1 | 4 | 0 |  |  |  |  |
| 2 | 4 | 0 |  |  |  |  |
| 3 | 4 | 2694 | 0.116 | 313 |  |  |
| 4 | 4 | 2324 | 0.116 | 269 |  |  |
|  |  | 5018 |  | 582 | 0.116 | 0.116 |
| All stages | | | | | | |
| Stage | Breast conserving surgery | Proportion of BCS | Mastectomy | Proportion of Mastectomy |  |  |
| DCIS | 4839 | 0.80 | 1210 | 0.20 |  |  |
| Stage I | 25640 | 0.92 | 2230 | 0.08 |  |  |
| Stage II | 20880 | 0.76 | 6694 | 0.24 |  |  |
| Stage III | 1009 | 0.116 | 7685 | 0.88 |  |  |
| Stage IV | 669 | 0.116 | 5094 | 0.88 |  |  |

**Stage-specific Survival Estimates from Munich Cancer Registry Reports**

Absolute survival rates were taken from Munich cancer registry(13). The survival rates for each year of diagnosis were calculated using life-table approach. The Munich cancer registry reported survival in subcategories according to the TNM staging. The categories are defined as T1N0M0, T2N0M0, T3N0M0, T4N0M0, T1NXM0, T2NXM0, T3NXM0, T4NXM0, T1N+M0, T2N+M0, T3N+M0, pT4N+M0, T_N_M1. The categories were converted to stages (Table S2, and Table A.6). We could not include T1NXM0, T2NXM0, T3NXM0, T1N+M0, and T2N+M0 because these subcategories fall in more than one TNM stage (14).

*Table S2. Stage conversion*

|  | **T1N0M0** | **T2N0M0** | **T3N0M0** | **T4N0M0** | **T4NxM0** | **T3N+M0** | **T4N+M0** | **M1*** |
| --- | --- | --- | --- | --- | --- | --- | --- | --- |
| **Stage I** | 1 |  |  |  |  |  |  |  |
| **Stage II** |  | 1 | 1 |  |  |  |  |  |
| **Stage III** |  |  |  | 1 | 1 | 1 | 1 |  |
| **Stage IV** |  |  |  |  |  |  |  | 1 |

***Table A.6. Stage-wise absolute survival estimates.***

| **Year** | **Stage I** | **Stage II** | **Stage III** | **Stage IV** |
| --- | --- | --- | --- | --- |
| **1** | **0.057** | **0.098** | **0.113** | **0.357893** |
| **2** | **0.085** | **0.151** | **0.201** | **0.550793** |
| **3** | **0.116** | **0.205** | **0.277** | **0.684067** |
| **4** | **0.153** | **0.253** | **0.346** | **0.75501** |
| **5** | **0.19** | **0.303** | **0.399** | **0.805583** |
| **6** | **0.226** | **0.347** | **0.444** | **0.8539** |
| **7** | **0.263** | **0.39** | **0.479** | **0.873919** |
| **8** | **0.299** | **0.426** | **0.508** | **0.885777** |
| **9** | **0.334** | **0.457** | **0.533** | **0.894789** |
| **10** | **0.373** | **0.484** | **0.555** | **0.900887** |
| **11** | **0.401** | **0.508** | **0.58** | **0.905496** |
| **12** | **0.432** | **0.534** | **0.601** | **0.907058** |
| **13** | **0.46** | **0.564** | **0.619** | **0.910156** |
| **14** | **0.493** | **0.598** | **0.639** | **0.914762** |
| **15** | **0.519** | **0.616** | **0.655** | **0.920848** |

**S3 – Cost of Breast Cancer in Germany**

**Cost of illness (Federal Statistical Office)**

In the reported healthcare expenditure statistics for the years 2015, the Federal Statistical Office Germany (Statistisches Bundesamt) reported a total healthcare expenditure of 338,207 million euros. The Federal Statistics Office only reported spending for prevention, inpatient, outpatient, and emergency services. The authorities collect overall expenditure and use a top-down approach to report the cost for each health disorder. Among the reported direct medical costs, cancers are the fifth leading cause of health expenditure and cost approximately 6.8% (23,002 million euro) of total healthcare expenditures. Analysing the cancer cost separately, breast cancer is the first leading cause of cancer expenditure; it consumed about 9.4% of the total cancer cost. BC is a female associated disease. However, less than 1% of BC related health expenditure was associated with male patients as well. The breakdown of the breast cancer cost is given in table A3.

***Table A.7. Total medical cost of BC in millions of euro for German female population in 2015.***

| **Component** | **Cost** |
| --- | --- |
| Public health | 6 |
| Office of Physicians | 236 |
| Office of Dentists | 0 |
| Office of health practitioners | 162 |
| Dispensing chemist | 282 |
| Retail sale | 33 |
| Home healthcare services | 204 |
| Hospitals | 652 |
| Preventive care /Rehabilitation services | 197 |
| Stationary /semi stationary nursing homes | 172 |
| Ambulance services | 7 |
| Administration | 114 |
| Rest of the world | 4 |
| Sostige Einrichtungen und private Haushalte | 81 |
| **Total** | **2150** |

**S4 – Sensitivity analysis**


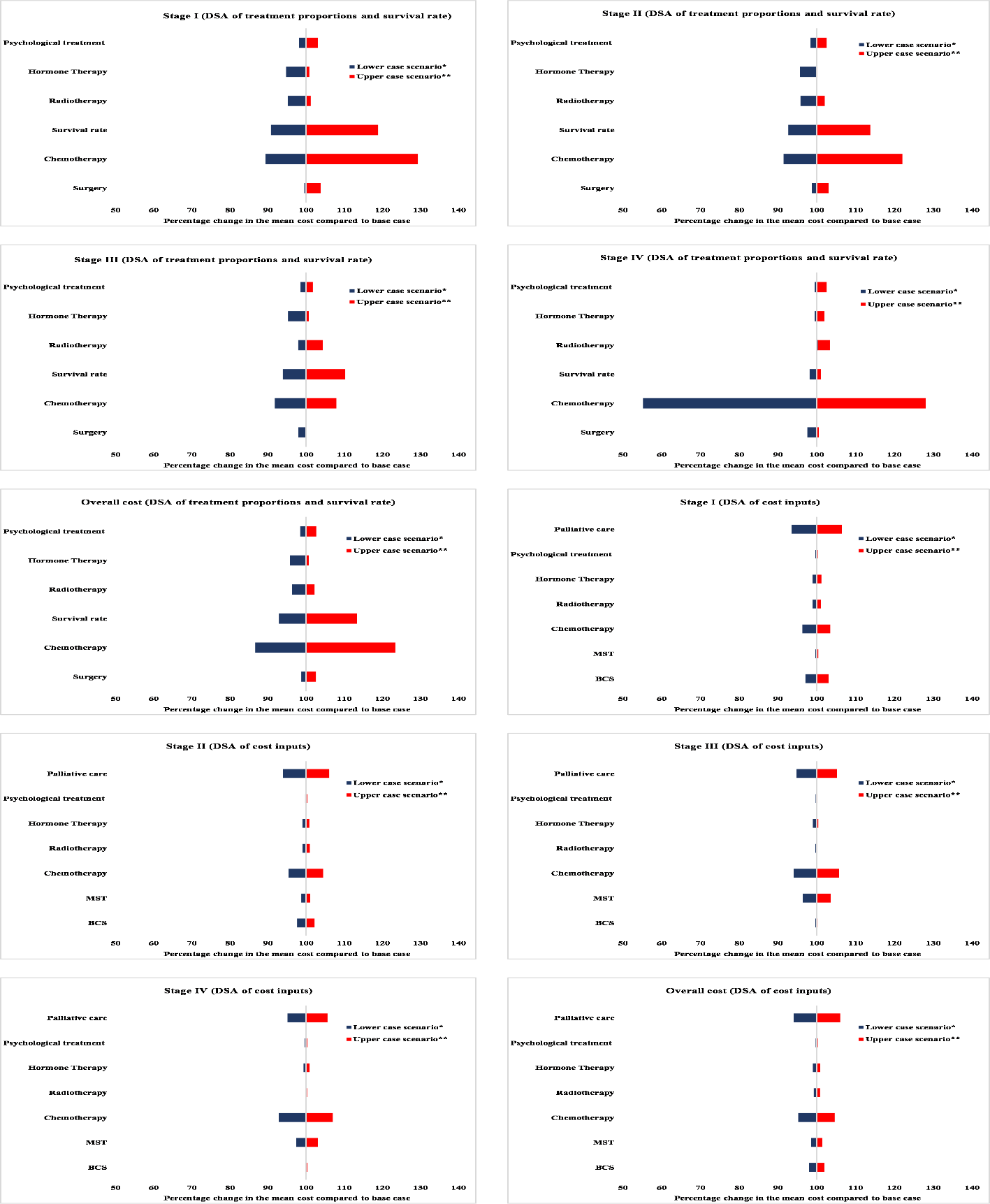


***Figure A.2*. *Deterministic sensitivity analysis of variations in assumed proportion of treatment scenarios and the unit cost parameters.***

DSA= Deterministic sensitivity analysis, DCIS= Ductal carcinoma in situ, BCS= Breast conserving surgery, MST= Mastectomy.
The y-axis shows the parameters and the x-axis shows the percentage change in the mean treatment cost when each parameter is changed according to the defined scenarios.

Source: Authors' elaboration

**References:**

1. Liberati A, Altman D, Tetzlaff J, Mulrow C, Gotzsche P, Ioannidis JJB. The Prisma Statement for Reporting Systematic Reviews and Meta-Analyses of Studies That Evaluate Healthcare Interventions: Explanation and Elaboration. (2009) 339:b2700-b.

2. Hamada S, Hinotsu S, Ishiguro H, Toi M, Kawakami K. Cross-National Comparison of Medical Costs Shared by Payers and Patients: A Study of Postmenopausal Women with Early-Stage Breast Cancer Based on Assumption Case Scenarios and Reimbursement Fees. *Breast care (Basel, Switzerland)* (2013) 8(4):282-8. Epub 2014/01/15. doi: 10.1159/000354249.

3. Schrauder MG, Brunel-Geuder L, Häberle L, Wunderle M, Hoyer J, Reis A, et al. Cost-Effectiveness of Risk-Reducing Surgeries in Preventing Hereditary Breast and Ovarian Cancer. *Breast (Edinburgh, Scotland)* (2017) 32:186-91. Epub 2017/02/20. doi: 10.1016/j.breast.2017.02.008.

4. Müller D, Danner M, Rhiem K, Stollenwerk B, Engel C, Rasche L, et al. Cost-Effectiveness of Different Strategies to Prevent Breast and Ovarian Cancer in German Women with a Brca 1 or 2 Mutation. *Eur J Health Econ* (2018) 19(3):341-53. Epub 2017/04/07. doi: 10.1007/s10198-017-0887-5.

5. Blank PR, Filipits M, Dubsky P, Gutzwiller F, Lux MP, Brase JC, et al. Cost-Effectiveness Analysis of Prognostic Gene Expression Signature-Based Stratification of Early Breast Cancer Patients. *PharmacoEconomics* (2015) 33(2):179-90. Epub 2014/11/19. doi: 10.1007/s40273-014-0227-x.

6. Lux MP, Reichelt C, Karnon J, Tänzer TD, Radosavac D, Fasching PA, et al. Cost-Benefit Analysis of Endocrine Therapy in the Adjuvant Setting for Postmenopausal Patients with Hormone Receptor-Positive Breast Cancer, Based on Survival Data and Future Prices for Generic Drugs in the Context of the German Health Care System. *Breast care (Basel, Switzerland)* (2011) 6(5):381-9. Epub 2012/05/24. doi: 10.1159/000333118.

7. Gruber EV, Stock S, Stollenwerk B. Breast Cancer Attributable Costs in Germany: A Top-Down Approach Based on Sickness Funds Data. *PloS one* (2012) 7(12):e51312. doi: 10.1371/journal.pone.0051312.

8. Kreis K, Plöthner M, Schmidt T, Seufert R, Schreeb K, Jahndel V, et al. Healthcare Costs Associated with Breast Cancer in Germany: A Claims Data Analysis. *Eur J Health Econ* (2020) 21(3):451-64. Epub 2020/01/04. doi: 10.1007/s10198-019-01148-w.

9. Arnold M. Simulation Modeling for Stratified Breast Cancer Screening – a Systematic Review of Cost and Quality of Life Assumptions. *BMC Health Serv Res* (2017) 17(1):802. doi: 10.1186/s12913-017-2766-2.

10. Arnold M, Pfeifer K, Quante AS. Is Risk-Stratified Breast Cancer Screening Economically Efficient in Germany? *PloS one* (2019) 14(5):e0217213. Epub 2019/05/24. doi:

10.1371/journal.pone.0217213.

11. Heerey A, McGowan B, Ryan M, Barry M. Microcosting Versus Drgs in the Provision of Cost Estimates for Use in Pharmacoeconomic Evaluation. *Expert review of pharmacoeconomics & outcomes research* (2002) 2(1):29-33. Epub 2002/02/01. doi: 10.1586/14737167.2.1.29.

12. Schrodi S, Niedostatek A, Werner C, Tillack A, Schubert-Fritschle G, Engel J. Is Primary Surgery of Breast Cancer Patients Consistent with German Guidelines? Twelve-Year Trend of Population-Based Clinical Cancer Registry Data. *European journal of cancer care* (2015) 24(2):242-52. Epub 2014/04/02. doi: 10.1111/ecc.12194.

13. München T. Icd-10 C50: Mammakarzinom (Frauen)—Survival. *München: Tumorregister München* (2021).

14. Koh J, Kim MJ. Introduction of a New Staging System of Breast Cancer for Radiologists: An Emphasis on the Prognostic Stage. *Korean J Radiol* (2019) 20(1):69-82. Epub 2018/12/27. doi: 10.3348/kjr.2018.0231.
